# Research on the Damage of Adrenal Pheochromocytoma to Patients' Cardiovascular Vessels and Its Correlation with Hypertension

**DOI:** 10.1155/2022/3644212

**Published:** 2022-02-11

**Authors:** Xiaohui Wang, Qiuping Zhao, Haiqiang Sang, Jiajia Dong, Minfu Bai

**Affiliations:** ^1^Department of Hypertension Control Center, Henan Provincial People's Hospital, People's Hospital of Zhengzhou University, Zhengzhou, Henan 450003, China; ^2^Department of Hypertension, Henan Provincial People's Hospital, People's Hospital of Zhengzhou University, Zhengzhou, Henan 450003, China; ^3^Department of Cardiology, The First Affiliated Hospital of Zhengzhou University, Zhengzhou, Henan 450000, China

## Abstract

Chromaffin cell-centered pheochromocytoma (Pheo) is a rare tumour. Pheochromocytoma and how it affects the heart will be the topic of this article. Due to the comparable symptoms and indications of the sympathetic nervous system, a pheochromocytoma might be difficult to detect early. There are also other frequent differential diagnoses that might delay the detection of a pheochromocytoma. One of the most common side effects of pheochromocytoma is unmanageable hypertension. Hypertensive crisis (extreme increases in blood pressure) can develop, which is a life-threatening condition that leads to strokes or arrhythmia. Estimated to affect African Americans significantly, they frequently go undetected due to a lack of resources or accessibility of services. Because this tumour is difficult to identify and its symptoms often resemble those of other diseases, it is frequently overlooked. A pheochromocytoma's long-term consequences can include cardiac muscle deterioration, congestive heart failure (CHF), a higher diabetes risk and possibly death. Masked hypertension (MH) is more common in people with adrenal pheochromocytoma, which has been related to an increased risk of heart disease. With the use of ambulatory blood pressure monitoring, this research set out to find out how common mental health issues are among people with APs. There were 85 participants in all, 43 of whom had APs and 42 of whom had the same age, gender, BMI, smoking and diabetes as the AP patients. Measurements were made of the BP and AP in both the diseased and control groups. Retrospective data collection was used to gather biochemical, hormonal and radiological information on the patients. The Pearson–Boltzmann CNN method was then used to assess risk based on the diagnosis results. Furthermore, depending on the risk score, more nonselective blockers (e.g., prazosin, doxazosin, terazosin, and metyrosine) have been used to lower perioperative catecholamine levels, hence reducing illness risk. After a successful surgical excision of the tumour, the recommended therapy can usually be stopped quickly.

## 1. Introduction

In the extra-adrenal paraganglia or in the medulla of the adrenal gland, chromaffin cells develop into cancerous tumours called pheochromocytomas. When it comes to sympathetic paragangliomas, which arise from the pheochromocytoma, only 23% of them produce norepinephrine or epinephrine and release it into the body. Pheochromocytomas are cancers of the adrenal glands and tumours of the sympath paraganglia. Pheochromocytomas occur in 0.2%–0.6% of people with hypertension. As a result, pheochromocytoma diagnostic workup is hampered by clinical symptoms that are indistinguishable from those of idiopathic hypertension, hyperthyroidism, heart failure and headaches. About 95% of pheochromocytoma patients experience hypertension as a result of catecholamine excesses. The clinical manifestations of hypertension can be long-lasting or short-lived. Hypertensive paroxysms may occur in certain individuals, despite the presence of a steady bp. Pheochromocytoma patients, on the other hand, account for a small but significant percentage of those diagnosed. The underlying pathophysiologic mechanisms of pheochromocytoma-related hypertension are examined in the following section. It is possible for patients with pheochromocytoma to also suffer from headaches and palpitations. Hypertension caused by pheochromocytoma was significantly connected to the Catecholamine Biosynthesis. Catechumin-positive secretory granules may also be seen in the parasympathetic paraganglia in the adrenal gland or autonomic paraganglia, which are both home to chromaffin cells that manufacture and release catecholamines. The enzyme machinery of catecholamine-producing cells is critical to the generation of catecholamines. A catecholamine-producing cell enzyme known as tyrosine hydroxylase (TH) converts L-tyrosine into dopa in the first step of the process. A negative feedback loop is created when tertahydropteridine (TH4) is oxidised by catecholamines, which generally co-factors with molecular oxygen and stops TH4 from operating. A co-factor for aromatic L-amino acid decarboxylase (AADC) is pyridoxal phosphate, which decarboxylates L-dihydroxyphenylethylamine (dopamine). The dopamine-hydroxylase (DBH) in the neurosecretory vesicles subsequently hydroxylates dopamine to L-norepinephrine. Norepinephrine is converted into epinephrine by the enzyme phenylethanolamine-N-methyl transferase (PNMT). While pheochromocytoma is an excellent mimic, it is also one of the most intriguing clinical paradoxes, with symptoms and indications that vary to nearly incomprehensible degrees in clinical circumstances that look identical. There are several pathophysiologic elements that contribute to clinically significant challenges to understand this paradox. Some of these factors, such as, for instance, are as follows:

- In health and sickness, catecholamine production in several chromaffin cell organs
- Variations in the catecholamine synthesis enzymatic machinery
- Substrate availability
- The size and quantity of secreting tissue, as well as its metabolic functions
- Catecholamine secretion type
- The amount of catecholamine secreted
- Catecholamine secretion pattern
- End-organ injury and a slew of other factors

To understand pheochromocytoma clinically, a detailed understanding of catecholamine synthesis and metabolism is essential. The purpose of this study was to determine the prevalence of hypertension in patients with pheochromocytoma. The rest of the paper may be broken down into the following sections: Section 1 introduces hypertension and pheochromocytoma in a simple manner.  Section 2 depicts the relevant current approaches.  Section 3 illustrates the problem definition. Analysis of hypertension associated with pheochromocytoma is discussed in Section 4, while the results and interpretations are discussed and the findings are summarized in Section 5.

## 2. Related Works

In [1], resistance to high blood pressure must be achieved in patients with hypertension and metabolic alkalosis by balancing the plasma aldosterone concentration (PAC) and plasma renin activity (PRA). In patients with hypertension and hypokalemia, the systolic and diastolic blood pressure will be of 160 mm Hg systolic or 100 mm Hg diastolic along with high plasma renin activity observed. Aldosterone suppression testing, which may be done either orally or intravenously, should be performed in the event of a positive PAC/PRA ratio result. In [2], it is addressed that those with PPGL and primary hypertension have a different steroid synthesis than those with normal levels of the hormones. In [3], toxic hypertension may result from pheochromocytoma, an adrenal gland tumour that produces catecholamines. In pregnancy, it affects just 0.1%–0.5% of hypertensive persons (0.0018–0.006 percent). There are a number of medications that might cause a patient's health to deteriorate, such as those often prescribed to pregnant women. In [4], there is a possibility that 5%–15% of persons with hypertension have an underlying etiology that is possibly treatable. Pheochromocytoma (PCC), an uncommon cause of hypertension that affects only 0.2% of all hypertensive individuals, is one of these reasons. In [5], it is focused on the endocrine etiology of secondary hypertension, including pheochromocytomas (PCCs) and paragangliomas as examples (PGLs). Hypertension is a symptom of around 15 endocrine diseases. Rare but possibly life-threatening causes are among the PCCs and PGLs. A prompt diagnosis and referral can save a person's life. We describe a method for screening and diagnosing these patients in this paper, with a focus on the necessity of genetic analysis. In [6], the authors give a complete assessment of the numerous cardiovascular symptoms and consequences of PPGLs that have been documented. In [7], patients with malignant pheochromocytoma are proven to have increased VEGF levels in their blood, showing that VEGF is released by the tumour. It is important to point out that malignant pheochromocytoma is not always accompanied by hypertension, as this case study has shown. In [8] followed by selective 1-adrenergic blockers such as doxazine/prazozine/terazosin (i.e., propranolol, atenolol). As a first line of defence against an increase in blood pressure, blocking peripheral-adrenergic receptors is never recommended. Labetalol has long been regarded the best agent because of its adrenergic antagonistic properties despite the fact that experimental data do not support its usage in this clinical setting. The use of calcium channel blockers may be necessary to maintain blood pressure management in patients with hypertension. In [9], the surgical excision of a pheochromocytoma may demand specific postoperative care since this tumour is potentially dangerous. In [10], ECMO and medical therapy were employed to bridge the gap between a catecholamine-induced cardiogenic shock and the surgical removal of the adrenal gland. In [11], patients with PPGL who tested negative for urinary metanephrines were the primary focus of this investigation. It was hoped that variations in the presenting symptoms among those who had been tested for PPGLs might assist evaluate the risk of disease in this prospective observational cohort research. In [12], this article focuses on the three types of cardiomyopathy that might affect a PHEO patient. CICMPP patients' clinical appearance and diagnostic and therapeutic strategies are also included. In [13], clinically diagnosed with PCC/PGL in public Spanish hospitals, TMEM127, MAX, RET, SDHD, SDHC, SDHB, SDHA, SDHAF2 and VHL all were recommended to us for genetic analysis in the important PCC genetic variants. There were no patients with neurofibromatosis type 1 clinical characteristics. Using standard techniques, DNA was taken from blood samples from 447 seemingly unrelated index cases collected between 1995 and 2012, including 36 cases identified as paediatric cases. Each patient, or a legal tutor in the case of children, provided written informed permission. Except for the RET oncogene, thorough genetic characterisation of susceptibility genes involves exon and intron–exon boundary point mutation analysis, as well as assessment of substantial deletions using multiplex ligation-dependent probe amplification or multiplex PCR. In the case of RET, researchers looked into point mutations impacting exons 16, 15, 14, 13, 11 and 10. [14] The goal of this retrospective study was to look into pheochromocytoma (pheo), a rare endocrine tumour that affects children.

## 3. Proposed Work

**Figure 1:**
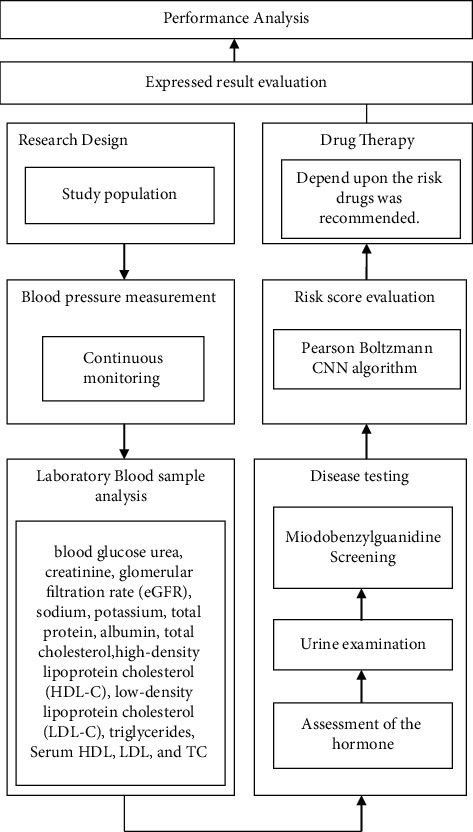
Schematic representation of the suggested methodology.

Norepinephrine, epinephrine and dopamine are catecholamines that act on G-protein associated adrenoceptors that are found throughout the body and are involved in practically every aspect of human biology. The *α*1, *α*2 and *β*1 receptors are stimulated by norepinephrine, whereas *β*1 and *β*2 denotes receptors that are only activated by epinephrine. Dopamine has no impact on any of the adrenergic receptors at normal dosages, but if the plasma level of dopamine rises (for example, in a dopamine-secreting tumour), dopamine may activate both adrenergic receptors simultaneously. 1-adrenergic receptors may elicit vasoconstriction when activated in several parts of the skeletal system, including the kidneys, the brain, the heart and many other arteries and veins. As a result, systemic pressure rises and organ perfusion falls. In cardiomyocytes, it likewise has a favourable inotropic effect (Figure 1).

### 3.1. Mechanism

**Figure 2:**
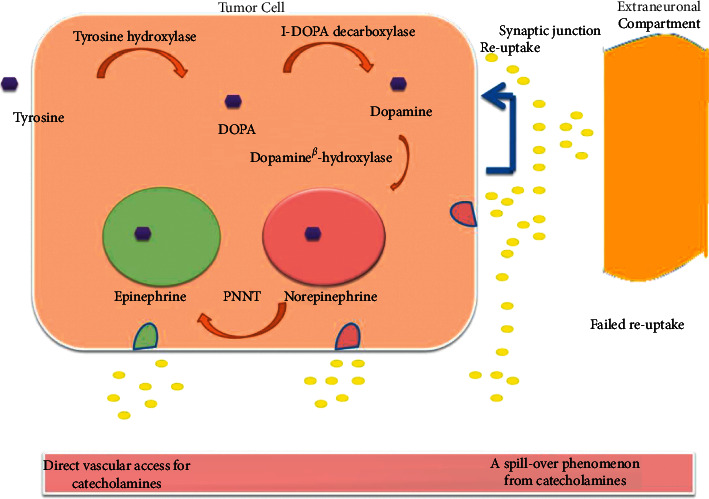
Disease mechanism.

2-adrenergic receptors on the presynaptic surface of sympathetic ganglia act as a negative feedback loop for the release of norepinephrine. Smooth muscle 2-adrenergic receptors are activated, resulting in an increase in blood flow to the arteries and a decrease in blood flow to the coronaries. The 1-adrenergic receptors can be stimulated by norepinephrine and epinephrine, both of which are neurotransmitters. The positive inotropic effect of 1 activation in cardiomyocytes is significantly greater than that of a 1 stimulus. As an additional benefit, activation of the heart's pacemaker results in an increase in lifespan. A rise in mean arterial pressure is caused by the release of renin from activated 1 receptors, which convert angiotensinogen into angiotensin I. Norepinephrine is released from the sympathetic ganglia in response to epinephrine stimulation of 2-adrenergic receptors. Dopamine will target the D1 and D2 dopaminergic receptors. Norepinephrine production from sympathetic nerve terminals is inhibited by D2 receptor activation, which has a mild inotropic effect on the pulse. Patients with dopamine-secreting pheochromocytomas may not have hypertension or palpitations because of the signalling net effect. Vasoconstriction and an increased heart rate can be caused by pharmacologically large amounts of dopamine, on the other hand. Treatment and diagnosis have long revolved around deadrenergicising adrenergic receptors. Both normal persons and patients with pheochromocytoma have been discovered to have significant desensitisation of their adrenal receptors. Clinical evidence shows that several people with pheochromocytoma-related hypercatecholaminemia remain asymptomatic despite little or no therapy. Keep in mind that the processes and rates of desensitisation vary from person to person; therefore, it is crucial to keep this in mind. Even in patients who have become somewhat desensitised, the rapid secretion of substantial amounts of catecholamines, known as paroxysmal hypercatecholaminemia, is likely to cause a significant clinical episode. When catecholamine levels start to rise again, receptor resensitisation seems to be reversible. Tumor dopamine, norepinephrine and epinephrine concentration varies according to enzyme activity. It has been discovered that pheochromocytomas contain significant amounts mRNA for the AADC, DBH and TH enzymes. Greater levels of catecholamine production are closely correlated with increased TH expression. Glucocorticoids, on the other hand, have a favourable effect on PNMT function in normal adrenal tissue. Increased activity of PNMT (glucocorticoids) in the cell may raise epinephrine levels. For some reason, although extra-adrenal tumours have PNMT incidence that is higher than in adrenal pheochromocytomas, the PNMT levels in these tumours are lower than those in normal adrenal tissue. As a therapeutic diagnostic tool, pheochromocytoma's secretory properties will be used. Most tumours that originate outside of the adrenal gland produce norepinephrine, but dopamine-secreting tumours are rare. Many endocrine tumours type 2 are associated with adrenal pheochromocytomas, which produce a mixture of epinephrine and norepinephrine (MEN 2). Von Hippel-Lindau disease tumours secrete only norepinephrine. The only site where dopamine-secreting tumours may be found is in the extra-adrenal pheochromocytomas. DBH expression is decreased in several cancers, which suggests that norepinephrine and dopamine production are decreased. Pheochromocytomas generate a variety of hormones and peptides, including adrenalomedullin, vasoactive intestinal polypeptide, ACTH, neuropeptide Y, endothelin-1, somatostatin, atrial natriuretic factor and parathyroid hormone-related peptide. An additional neuroendocrine product's vasoconstrictive or vasodilatory properties, together with hypercatecholaminemia in the bloodstream, would decide the clinical presentation. Hypertensive paroxysms may be caused by regular exercise (activity, posture changes) or tumour manipulation, and the pattern of catecholamine production from the tumour might be steady, episodic, or both. Catecholamine secretion by tumours cannot be predicted because of the lack of information on when and how much catecholamines will be released during each secretory episode. It seems that hypertension symptoms and phenotype are connected. Norepinephrine-producing tumours are more likely to cause hypertension, while epinephrine-producing tumours are more likely to cause paroxysmal and orthostatic hypertension. With dopamine-secreting tumours, most patients do not have high blood pressure (Figure 2).

### 3.2. Research Design

Single-center, retrospective and cross-sectional design were used in this study's research design. The study took place at the Guangdong Heart Hospital in China between 15 December 2020 and 15 March 2021. People with and without NFAIs were both included in this research. Clients in the NFAI team had OBP readings of less than 140/90 mmHg and more than 120/80 mmHg (BP (normal) and BP (high)), respectively, on a regular basis. Participants' demographic and clinical data were recorded in the study database. When it comes to NFAIs, 43 persons above the age of 18 who were scanned for other medical reasons and found to have nonmalignant adrenal neoplasms would be included. Clinical and hormonal assessments have previously deemed the patients to be nonfunctioning or nonaggressive. Noncontrast computed tomography imaging (10 Hounsfield units) or CT with delayed contrast absolute washout >60% were used in all instances to measure the size of the adenoma. AIs were ruled out as functioning after a series of hormone tests, according to current guidelines. A positive control of 42 healthy persons was constructed based on age, age distribution, BMI, hypertension, tobacco and alcohol misuse. Antihypertensive and antihypertensive history medication use, persistent myocardial infarction (mi) (congestive heart failure, macabre adiposity, energetic psychotic issues, thyroid problems, hyperthyroidism, obstructive sleep symptoms), performance enhancing drugs and oral contraceptives and main or metastases adrenal cancers are all considered risk factors for cardiovascular disease. It was also thrown out of the research for individuals with blood cortisol levels below 1.8 g/dL after taking 1 mg DST. In cases where there was a lack of data, patients who had not been sufficiently checked hormonally and those who had adipose tissue diameters between 10 and 40 mm were excluded from the research. Calculating body mass index (kg/m^2^) is a simple matter of multiplying one's weight (kg) by one's height (m^2^).

### 3.3. Biochemical Analysis

#### 3.3.1. Blood Pressure Measurement

According to the 2018 ESC/ESH recommendations for the therapy of arterial hypertension, the diagnosis of arterial hypertension was made. All participants' systolic and diastolic blood pressures were measured in the outpatient clinic using the same sphygmomanometer (OMRON M3 Comfort (HEM-7134-E), OMRON HEALTHCARE Co., Ltd. KYOTO, 617–0002 JAPAN). Participants were encouraged to abstain from drinking, smoking and coffee/tea for at least 30 minutes before to the blood pressure measurement. In a sitting position with the arm supported at heart level and a cuff size appropriate for patients, the patient's blood pressure was measured. At 5-minute intervals, OBP was measured twice in both arms. For both arms, two measurements were averaged, and the highest value was recorded as BP. According to the OBP measurement, optimal blood pressure was <120/80 mmHg, normal blood pressure was 120–129/80–84 mmHg, high normal blood pressure was 130–139/85–89 mmHg and hypertension was ≥140/90 mmHg.

#### 3.3.2. Ambulatory Blood Pressure Monitoring

Only the patient's nondominant arm was subjected to ABPM using the Oscar II device from Suntech (USA, Morrisville, NC). Recording their sleep and wake times was instructed to the patients. Before ABPM, they were told to stay away from strenuous physical activity. ABPM was done 30 times a night and 15 times a day when the subjects were awake (sleep). At rest, the patient has a BP of 130/80 mmHg, while during the day, it is 135/85 mmHg and at night, it is 120/70 mmHg [12]. In contrast to the OBP of 141/91 mmHg, the MH is defined as an average of 131/81 mmHg over the course of 24 hours, with a daytime BP of 135/85 mmHg and a nighttime blood pressure of 121/71 mmHg. According to the results of the ABPM study, participants with NFAIs were divided into two groups: MH-positive and MH-negative.

#### 3.3.3. Laboratory Analysis

After a 12-hour fast, all participants had their venous blood samples obtained. The patients' fasting blood glucose, urea, creatinine, estimated glomerular filtration rate (eGFR), sodium, potassium, total protein, albumin, total cholesterol, high-density lipoprotein cholesterol (HDL-C), low-density lipoprotein cholesterol (LDL-C) and triglyceride levels were all measured. An automated clinical chemistry analyzer was used to measure HDL, LDL and TC values in the blood (Abbott Architect c16000). The CKD-Epidemiology Collaboration (CKD-EPI) was used to determine the eGFR. A Roche Cobas 8000 automated analyzer was used to measure biochemical parameters (Roche Diagnostics, Shanghai, Ltd.).

#### 3.3.4. Hormonal, Urinary and Radiological Assessments

The availability of NFAIs was assessed using the ESC Clinical Practice Guidelines as a guideline. Clinical signs and symptoms of primary hyperaldosteronism, Cushing syndrome, sex hormone–producing adenoma, pheochromocytoma and malignancy were assessed in all patients. All patients had their morning cortisol (g/dL), adrenocorticotropic hormone (ACTH, pg/mL), dehydroepiandrosteronesulphate, plasma renin activity (ng/mL per hour), plasma aldosterone (ng/dL) and 24-hour urine metanephrine/normetanephrine (*μ*g/day) levels tested. Assays were performed on urine samples. Miodobenzylguanidine Screening was started here for the purpose of radiological screening. In the study, screening with I-mIBG imaging was linked to a 21% reduction in ICD utilization, resulting in a screening number of 5 to prevent 1 ICD placement. In comparison to no screening, screening lowered expenses per patient by $5500 and $13,431 (in 2013 dollars) over 2 and 10 years, respectively, and resulted in losses of 0.001 and 0.040 life-years, respectively. Over two and ten years, screening was cost-effective, with savings of US ($5,248,404 and $513,036) per quality-adjusted life-year lost, respectively. Patients with an ejection fraction (EF) of 25%–35% saved more money in secondary outcomes than those with an EF of less than 25%.

### 3.4. Risk Score Evaluation

The Pearson–Boltzmann CNN technique was used to calculate the risk score based on the collected data. The ultimate purpose of the Pearson–Boltzmann CNN classification is to determine hypertension risk, indicating whether or not a person is at danger. When each anomalous data's posteriori probability is calculated. Comparing the symptoms of the disease to the disease forecast using the known real goal value by replicated processing, the classifier can make decisions about outcomes that include disease symptoms. The target value could be the known value for the sake of estimation. To lower the average squared error between the prediction and the true value, a weight set is updated for each datum. The results of the decision classifier used in the classifier are used to make these changes. The ranking process is sped up as a result of these revisions. Inputs, outputs and mistakes were among the parameters that were addressed in the steps. It can be expressed mathematically:

(1) $$\begin{array}{c} F\left(1\right)=W^{t}\left(l\right)+b, \end{array}$$

where *F*(l) is a functional matrix and *W* is the classifier of the dual variables.

After that, we need to discuss about the relationship among the data,

(2) $$\begin{array}{c} F\left(1\right)=\sum_{a_{i>0}}a_{i}y_{i}K\left(l_{i},X\right)+b. \end{array}$$

The feature error can be classification using the following equation:

(3) $$\begin{array}{c} \varphi \left(W\right)=-\emptyset {\left(W\right)}^{2}. \end{array}$$

Finally, a ranking is generated for the abnormality matching distance of base data.

(4) $$\begin{array}{c} {\text{obj}}_{\text{ED}}=-20*q\left(\frac{-2*\sqrt{\sum V_{v}}}{2}\right)-\exp\left(\sum \frac{\cos\left(2\pi *V_{v}\right)}{d_{b}}\right)+20\;\exp*\text{ED,} \end{array}$$

where the ED signifies the Euclidean distance, *q* denotes the query data and *s* is the signal score value.

(5) $$\begin{array}{c} \text{classify}\left(c\right)={\text{ED}}_{j}^{\text{l}}. \end{array}$$

The score classification was concluded as follows:

(6) $$\begin{array}{c} c_{d}=N{\left(\text{ED}\right)}_{j}^{l}\;-S{\left(\;E_{j}^{l}D_{j}^{l}\right)}^{2}\left(\frac{1}{H}\sum_{i}\sum_{j}\text{ED}\left(i,j\right)\cdot i^{2}\right). \end{array}$$

The procedure for the risk score evaluation is shown in Algorithm 1.

### 3.5. Drug Recommendation

Based on the risk rating, the client was prescribed one of four drugs: prazosin, doxazosin, terazosin and metyrosine.

#### 3.5.1. Prazosin

This drug is taken orally and has a rapid absorption rate; peak plasma concentrations are reached within one to two hours after ingesting it. Since it has a half-life of 2.5 h, it must be done twice or thrice a day. A particular cytochrome P450 involved in its metabolism has yet to be identified. Despite renal disease or aging, the pharmacokinetic of prazosin were unaffected.

#### 3.5.2. Terazosin

Terazosin is a long-acting, highly selective 1-receptor blocker in the treatment of BPH symptoms. Regardless of whether or whether it is taken with or without food, it is effectively absorbed. It takes an hour for plasma concentrations to reach their maximum. The peak plasma concentration is slowed by around 40 minutes when taken with meals. Due to its slow first-pass liver metabolism, the parent drug accounts for approximately all of the medication's circulating dose. One dosage of Terazosin each day is needed since it has a 12-hour half-life. Due to an aged population's decreased plasma clearance, terazosin's half-life is prolonged (approximately 14 hours).

#### 3.5.3. Doxazosin

As a result of its lengthy half-life of 16 hours, Doxazosin may be used once a day as a selective 1-receptor blocker. Take it with meal if you want it to work. Both young and elderly persons have the same oral clearance and plasma half-life. Due to the fact that it is metabolised in the liver, those with compromised renal function should be cautious while using it. Renal impairment has little effect on the pharmacokinetic characteristics of patients.

#### 3.5.4. Metyrosine

Tyrosine 3-monooxygenase inhibitor metyrosine is used in the treatment of pheochromocytoma with excessive sympathetic stimulation. Catecholamine synthesis is inhibited by tyrosine 3-monooxygenase inhibitor. Patients with pheochromocytoma who have too much sympathetic activity might benefit from this treatment.

## 4. Performance Analysis

**Figure 3:**
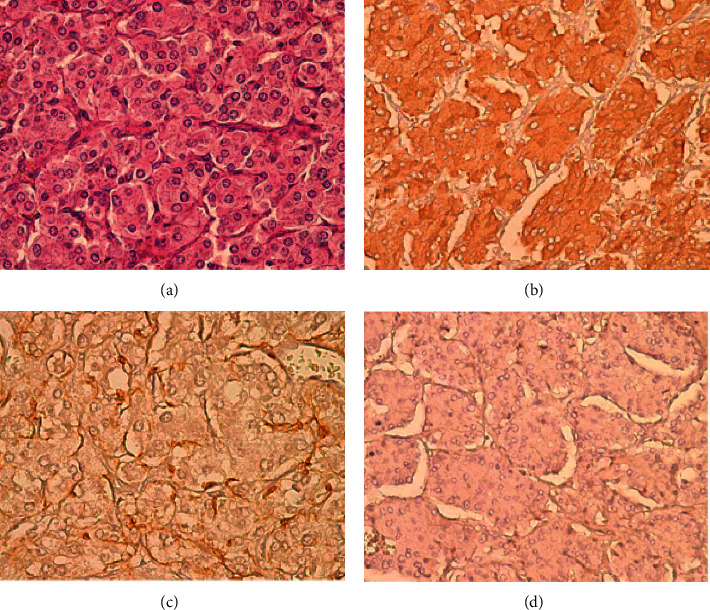
Clinical pathology of the high-risk score patients for (a) Patient 1; (b) Patient 2; (c) Patient 3; (d) Patient 4.

This section depicts a full explanation of the proposed system's performance analysis: a high-risk patient was recommended for experimentation (Figure 3).

Figure 3 represents the clinical pathology of the pheochromocytoma under laboratory condition.

**Figure 4:**
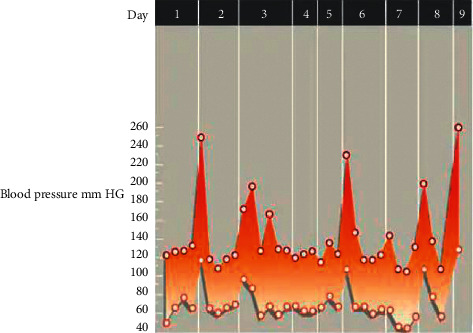
Pheochromocytoma and irregular blood pressure.

**Figure 5:**
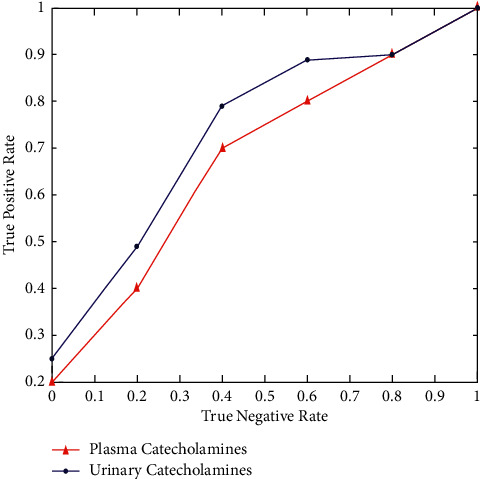
True negative vs. true positive.

The high-risk patient here has a tumour that emits hormones that can cause high blood pressure, headaches, sweating and panic attack symptoms (Figure 4). A pheochromocytoma can cause blood pressure to fluctuate wildly, with normal blood pressure in between. This makes it more difficult to diagnose the problem. Due to a pheochromocytoma, the graph depicts a nine-day period of small, unpredictable blood pressure spikes. The bottom number of the reading is represented by the lower points (diastolic pressure). The higher numbers represent the reading's highest number (systolic pressure). On day two, for example, a measurement of 250/110 millimetres of mercury indicates the first blood pressure spike (Figure 5).

**Figure 6:**
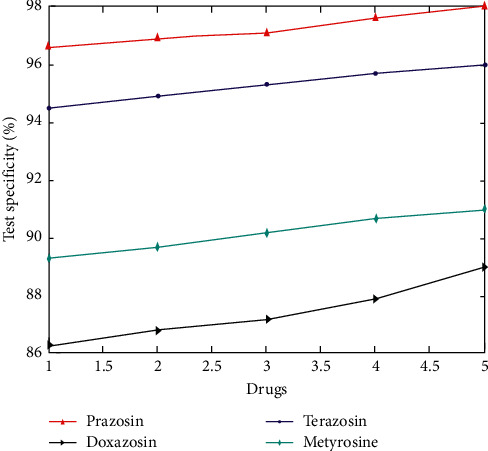
Drug specificity.

The negative predictive value of plasma and urine catecholamine testing at different pheochromocytoma prevalence revealed that a positive test result for the disease's state prediction was negative (Figure 6).

Table 1 shows the statistical analysis of the recommended drugs based on sensitivity and specificity.

Depending upon the drug, the production of the catecholamine was adjusted and the specificity values are obtained.

**Figure 7:**
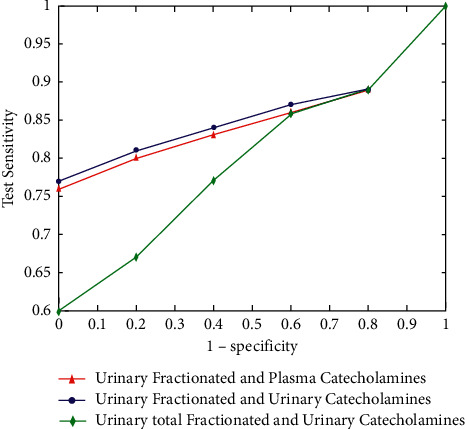
Sensitivity vs. specificity.

The relation between the specificity and the sensitivity of the catecholamine production was illustrated in Figure 7.

**Figure 8:**
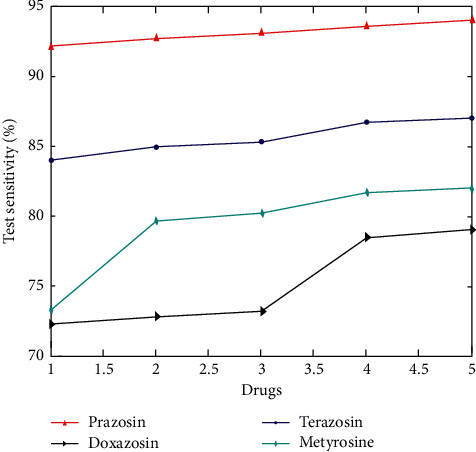
Drug sensitivity.

A positive or a negative result for the test of plasma or catecholamine changes the probability of having the pheochromocytoma [15–17] in relationship with the pretest and the posttest sensitivity probability (Figure 8).

**Figure 9:**
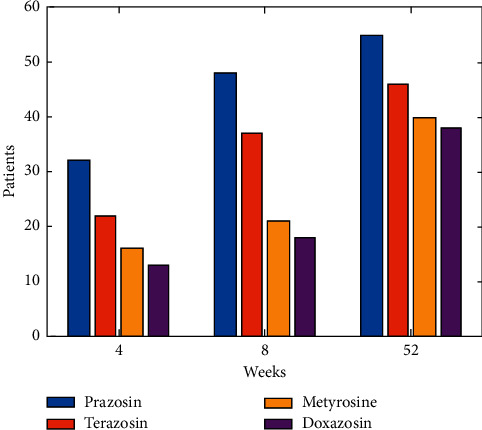
Patient condition improvement.

In Figure 9 terazosin, Doxazosin, Metyrosine and prazosin [18] groups, symptom ratings improved by 37.8% compared to 18.4% in the prazosin [19] groups. A clinically meaningful enhancement in prazosin was defined as a 35% decrease in the symptom score from baseline. Prazosin was shown to benefit 55% of patients compared to only 28% of those taking other medications at the previous visit.

## 5. Conclusion

Improved symptoms and quality of life, less bladder outlet blockage, better cholesterol profiles and lower blood pressure in hypertensive males are all good results of blocker medication. However, it might produce side effects like asthenia (10%), dizziness (10%), postural hypotension (50%) and syncope (4%) (0% to 1%). For optimal effectiveness and tolerance, 32-blocker medicine should be started at a low dosage, then gradually increased. After many days without a dosage of a -blocker, the medicine must be reproceeded with the original dosing schedule and titrated to therapeutic levels. Other antihypertensive drugs may have similar side effects as selective 1-blockers, which were originally used to treat hypertension. This means that -blocker side effects may occur as a consequence of lowering blood pressure. To understand how blood pressure is affected by the 1B and 1D subtypes, it is necessary to understand how vascular smooth muscle is mediated by these subtypes. Pheochromocytoma blood pressure changes were only shown to be identical with postural hypotension. Studies suggest that conventional -blockers' side effects are caused by processes other than blood pressure reduction and that the pace at which blood pressure drops present themselves may be more important than their quantity in producing unpleasant responses. Blocker tolerance may be influenced by other mechanisms, including as changes in how drugs enter the brain or how quickly they are excreted. Symptomatic enhancement in pheochromocytoma patients treated with -blockers has been associated to physiological deterioration, according to studies. As a consequence, we opted forprazosin, doxazosin, terazosin and metyrosine as preferred options. During follow-up investigations with these individuals, more than one symptom ratings and urine retention rates must be taken into account. Finding the root cause of pheochromocytoma and hypertension might help prevent long-term complications that may arise in certain patients.

## Figures and Tables

**Algorithm 1:**
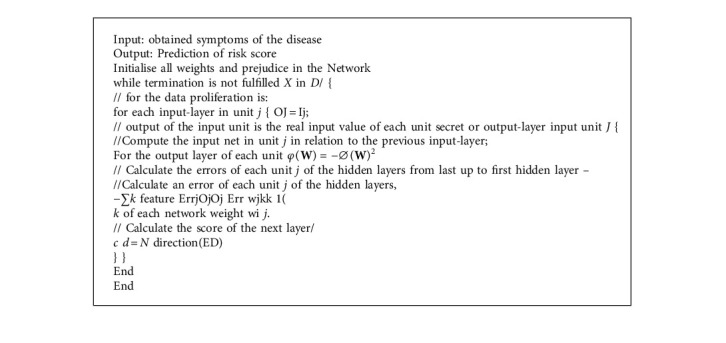
Pearson–Boltzmann CNN.

**Table 1:** Statistical analysis showing sensitivity and specificity for the recommended drugs.

Drug dosage	Recommended drugs							
	Prazosin		Terazosin		Doxazosin		Metyrosine	
	Sensitivity (%)	Specificity (%)	Sensitivity (%)	Specificity (%)	Sensitivity (%)	Specificity (%)	Sensitivity (%)	Specificity (%)

1	92	96.4	84	94.4	73	86.2	74	89.6
2	92.5	97	85	95	73.5	86.6	80	89.8
3	93	97.2	85.2	95.4	73.6	87	80.2	90
4	93.5	97.6	87	95.8	78	88	82	90.4
5	94	98	87.5	96	78.6	89	82.5	91

## Data Availability

The datasets used during the current study are available from the corresponding author on reasonable request.
